# Association of trichilemmal and basal cell carcinomas: a case report

**DOI:** 10.11604/pamj.2022.43.89.33114

**Published:** 2022-10-19

**Authors:** Moad El Mekkaoui, Bouchra Dani, Oussama Amraoui, Razika Bencheikh, Anas Benbouzid, Abdelilah Oujilal, Leila Essakalli, Hafsa Elouazzani, Ismail Boujida, Nadia Cherradi

**Affiliations:** 1 Service d'Oto-Rhino-Laryngologie et Chirurgie Cervico-Faciale, Centre Hospitalier Universitaire Ibn Sina, Rabat, Maroc,; 2 Laboratoire d'Anatomie Pathologique, Hôpital des Spécialités, Rabat, Morocco

**Keywords:** Trichilemmal carcinoma, basal cell carcinoma, actinic keratosis, case report

## Abstract

Proliferating trichilemmal tumor (PTT) is a benign tumor arising from the isthmic portion of the hair follicle. Malignant transformation in PPT is very rare and unusual. Indeed, only about sixty well-documented cases have been found in the English literature. We present here the case of a 72-year-old patient with an exceptional combination of malignant trichilemmal carcinoma and basal cell carcinoma, occurring on actinic keratosis lesions. The aim of this work is to describe the diagnostic and therapeutic modalities of this association which is exceptional.

## Introduction

Proliferating Trichilemmal Tumor (PTT) is a benign tumor arising from the isthmic portion of the hair follicle, which can be locally aggressive [1]. It is usually a solitary lesion involving the scalp in 90% of cases, but can also be found in other sites such as the forehead [2]. Its malignant transformation is very rare and unusual, usually evidenced by regional or distant metastasis [3,4]. Indeed, only about sixty cases have been found in the literature [5,6]. We will present an exceptional case associating an invasive malignant frontal trichilemmal carcinoma and a basal cell carcinoma occurring on actinic keratosis lesions, and we will explain the diagnostic, therapeutic and prognostic modalities of this entity.

## Patient and observation

**Patient information:** the patient was a 72-year-old man with no previous history of the disease. He presented to the emergency room of the Rabat Specialty Hospital with a swelling on the right forehead that had been growing for a year and was associated with other skin lesions on the right ear and face.

**Figure 1:**
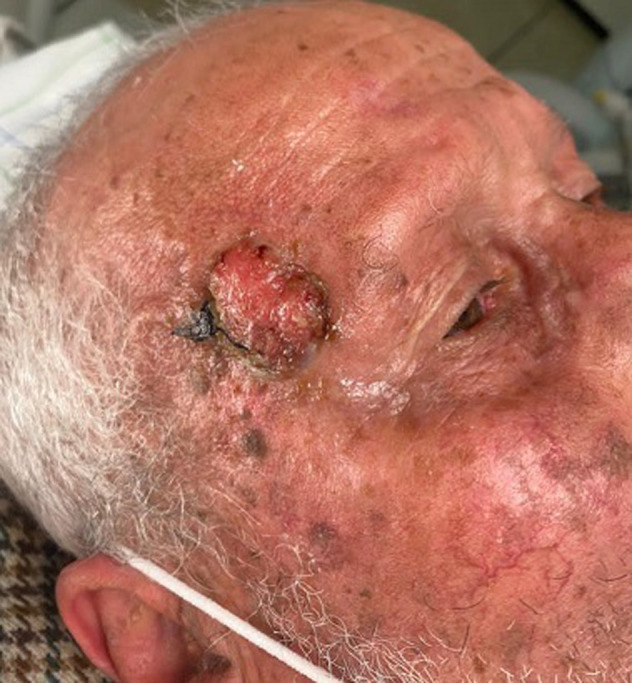
skin lesions of the face found

**Figure 2:**
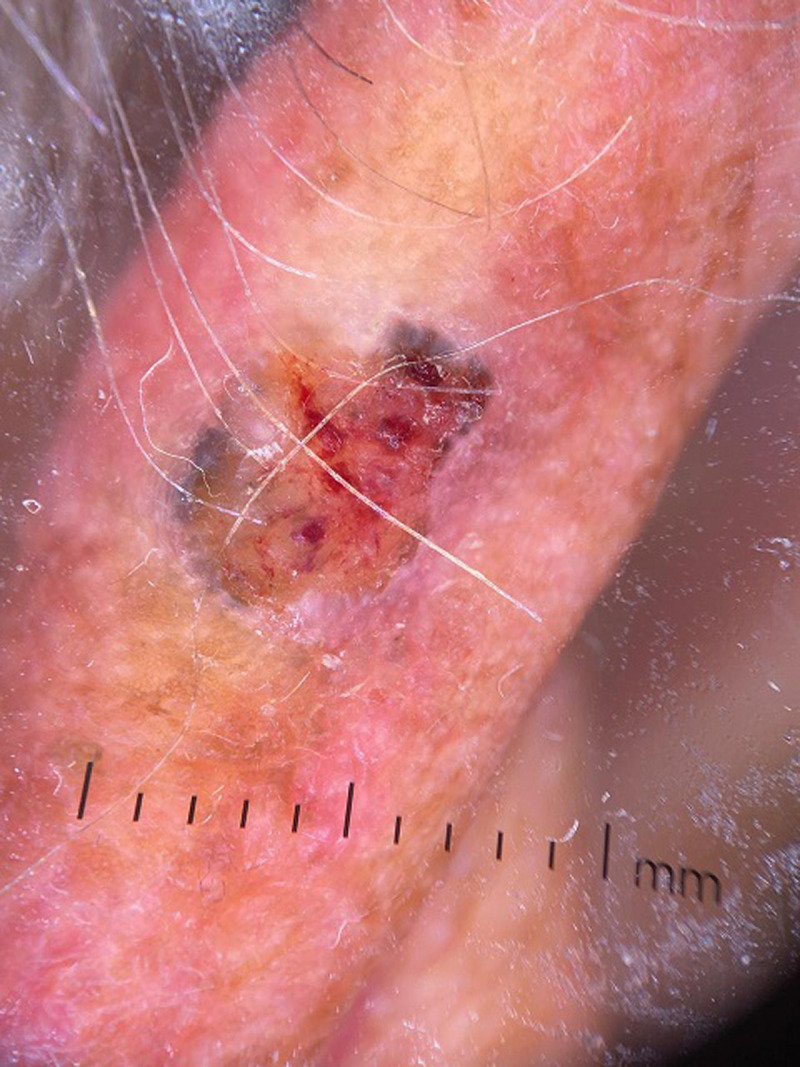
skin lesion of the right auricular pinna found

**Clinical findings:** clinical examination revealed a right frontal exophytic nodule 2 cm long, firm, mobile, painless and not ulcerated (Figure 1). Then, it revealed an ulcerated papule on the right auricle (Figure 2), and small patches of dry, crusted skin on the face (Figure 1). The lymph nodes were free.

**Diagnostic assessment:** dermoscopy suspected basal cell carcinoma of the right frontal and auricular area on actinic keratosis lesions well visible on the face. As part of the extension workup, a cervico-thoraco-abdomino-pelvic Computed Tomography (CT) scan was performed revealing no local or distant metastasis.

**Figure 3:**
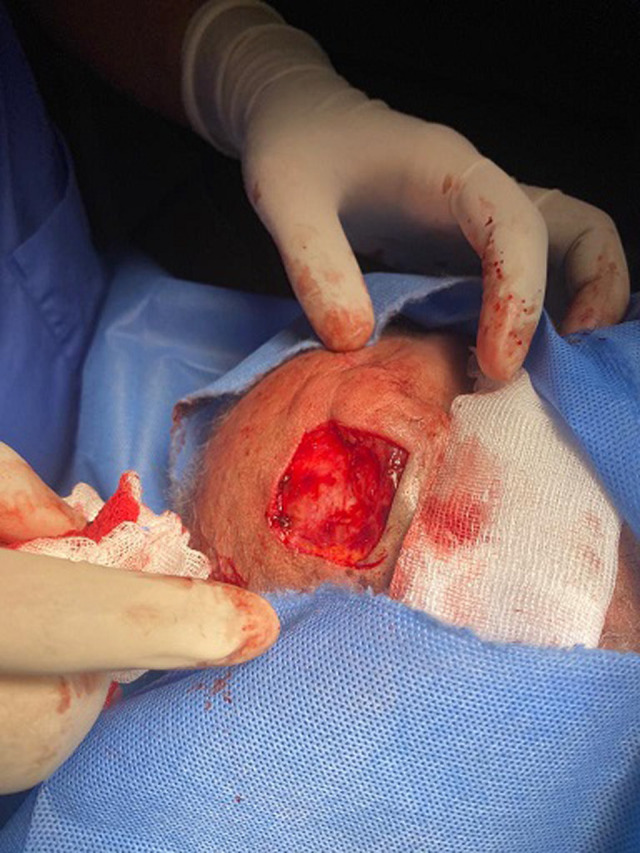
surgical removal of frontal lesion

**Figure 4:**
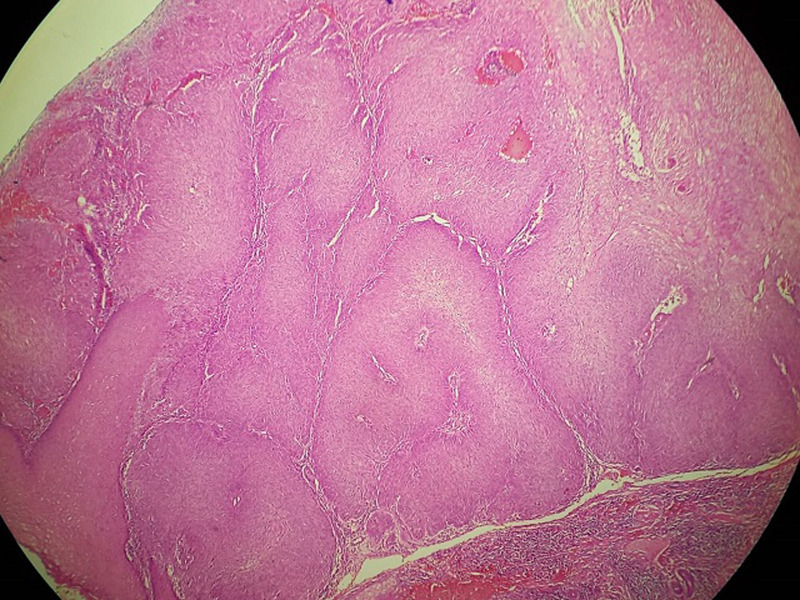
trichilemmal proliferation (HEX10)

**Figure 5:**
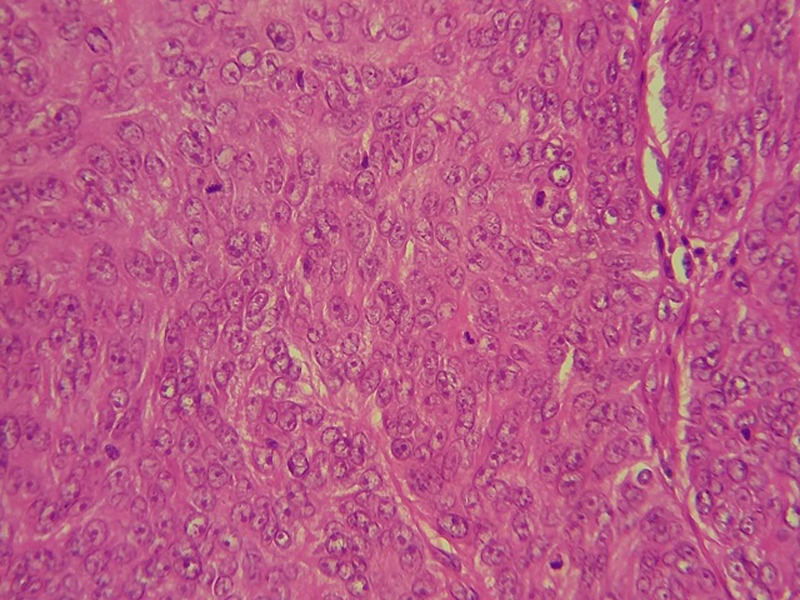
very important mitotic activity (HEX40)

**Figure 6:**
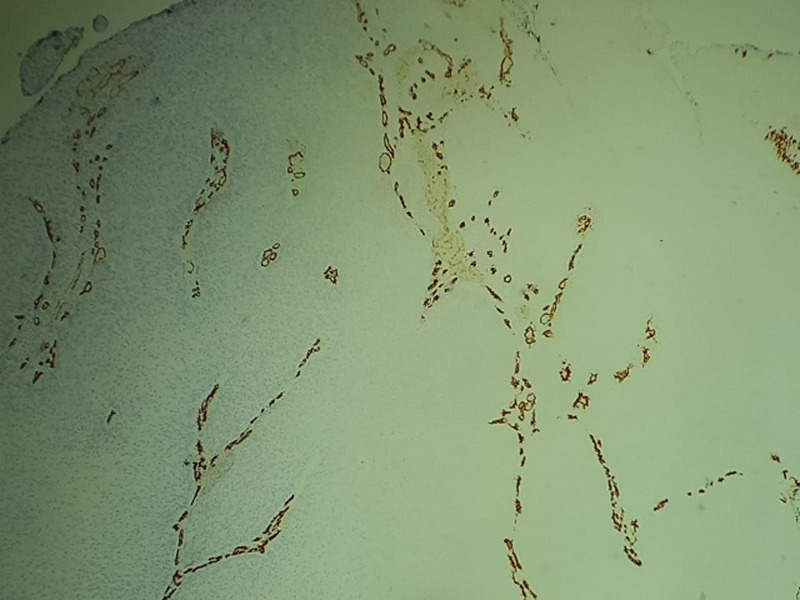
loss of CD 34 expression

**Figure 7:**
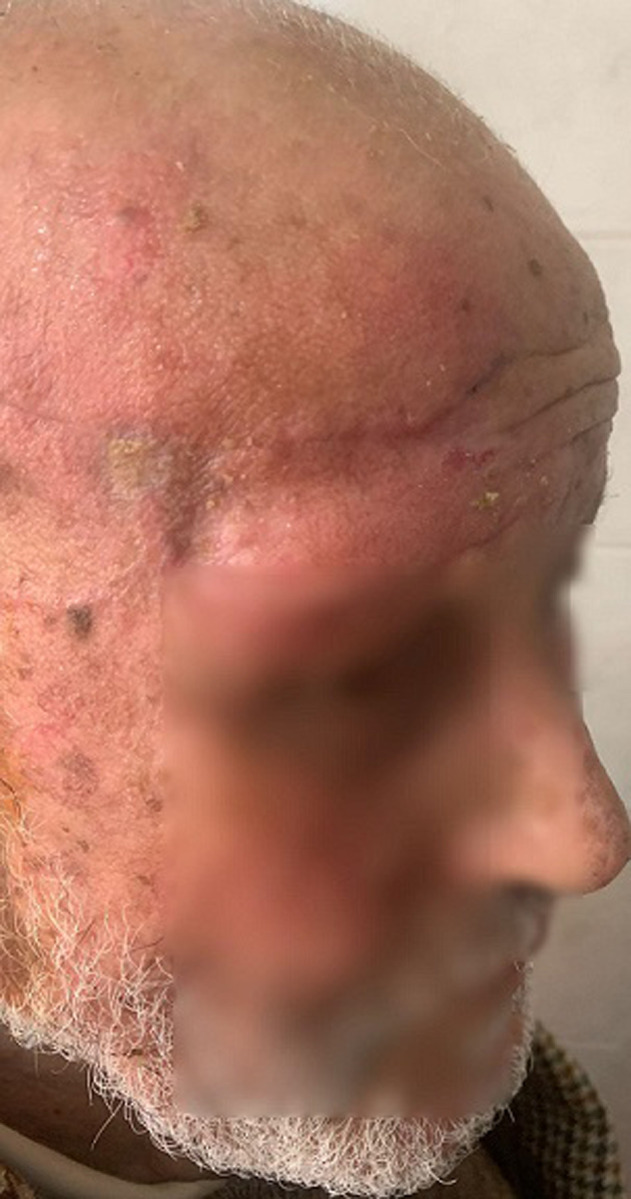
no local recurrence after 6 months

**Therapeutic intervention and follow-up:** the treatment consisted of a wide surgical excision of the right frontal swelling with an “H” shaped sliding flap of the forehead (Figure 3), associated with an excision of the right auricular skin lesion. The facial skin lesions were treated with cryotherapy. The specimens were fixed in 10% formalin. Anatomopathological and immunohistochemical examinations were in favour of a frontal trichilemmal carcinoma, selected in view of a trichilemmal proliferation made of endophytic cells centred by clear cells (HEX10) (Figure 4), a very marked mitotic activity (HEX40) (Figure 5), an absence of BEREP4 expression and a loss of CD 34 expression (Figure 6). While the right ear lesion was diagnosed as basal cell carcinoma. The surgical margins were healthy by 0.4 cm. Post-operative follow-up was 6 months, with no recurrence or metastasis (Figure 7).

**Informed consent:** written informed consent was taken from the patient to publish this case report.

## Discussion

Proliferating trichilemmal tumor (PTT) is a benign cutaneous adnexal tumor arising from the outer root sheath of a hair follicle and can be locally aggressive [1,2]. Malignant transformation is exceptional, as evidenced by regional or distant metastasis [2,3]. Indeed, malignant proliferating trichilemmal tumor (also called trichilemmal carcinoma) was first described by Saida in 1983 [4], due to a PTT that showed infiltrative growth, marked cytological atypia, high mitotic activity including atypical forms and lymph node metastasis [2,5]. Since then, only about sixty well-documented cases have been published in the literature, [2] some of which are associated with lymph node metastasis and more rarely hematogenous metastasis [3,5,6].

These tumors usually occur on sun-exposed areas of the elderly, favored by the presence of actinic keratosis lesions [7-9]. They occur on the scalp in 90% of cases, but can also be found on the forehead, nose, back, chest, abdomen, buttocks, elbow, wrist, pubic bone and vulva [1]. Most patients were female (84%) in the sixth and seventh decades of life [2]. In our case, it was a 72-year-old sun-exposed man with trichilemmal carcinoma and basal cell carcinoma on actinic keratosis lesions. A literature search was performed using the PubMed search engine using the keywords “carcinoma”, “trichilemmal” and “basal cell”. No cases reporting an association of these two entities were found.

Clinically, trichilemmal carcinoma presents as a solitary indolent lesion [9], which may be confused with squamous cell carcinoma, basal cell carcinoma, nodular melanoma or keratoacanthoma [9]. The diagnosis is established by histopathological examination using hematoxylin-eosin staining, which, if necessary, is supplemented by immunohistochemistry of the lesions [9]. The criteria for malignancy have been widely discussed in the literature. Indeed, marked cellular atypia associated with high mitotic activity can be observed in benign PTT. Conversely, typical cases of histologically quiescent PTT had a fatal course [6,10]. Thus, it is currently accepted that only the presence of frank stromal infiltration confirms malignancy [3,5,6]. According to Mehregan, the rapid and sudden development of PTT is a clinical predictor of malignancy [6,10]. Mones and Ackerman point out that an asymmetric appearance of the lesion induced by confluence and variation in size and shape of cell lobules is a definite criterion for malignancy even in well-circumscribed lesions. In addition, nuclear pleomorphism, mitoses, and cellular overlap are also cytological arguments in favor of malignancy [6]. The extension work-up should systematically include a cervico-thoraco-abdomino-pelvic CT scan given the risk of developing local cervical lymph node metastasis or distant hematogenous metastasis. The latter have rarely been reported in the medical literature [9], our patient did not have metastasis either.

The recommended treatment is complete surgical excision with verification of the surgical margins [6]. Incomplete excision should always lead to revision surgery. Histologically clear margins are crucial for a locally aggressive growth pattern and potential for local recurrence [9]. Radiotherapy with or without chemotherapy is reserved for advanced metastatic cases. Recurrence is common despite adequate resection with healthy surgical margins. Boscaino reported that after excision of lesions in seven patients, there was no tumor recurrence during a follow-up of 2 months to 4 years [8,9]. De Bin Xu, in a study of 26 CT cases between 1998 and 2012 with a mean follow-up of 63.8 months, showed that wide local excision with tumor-free margins yielded satisfactory results in 24 patients, while two patients had recurrence [9]. Thus, surgery is considered the treatment of choice for CT and periodic surveillance without adjuvant therapy is usually sufficient [9]. In case of metastasis, systemic chemotherapy should be considered (four cycles of cisplatin and vindesine or four cycles of cisplatin and cyclophosphamide) with the aim of controlling disease progression [9]. In our case, surgical excision was performed, the surgical margins were healthy 0.4 cm. The post-operative follow-up was 6 months, without recurrence or metastasis.

## Conclusion

In conclusion, this patient presented a combination of trichilemmal and basal cell carcinoma which was treated surgically with a good post-operative evolution. This exceptional association poses a diagnostic problem and therapeutic difficulties. These carcinomas generally occur on actinic keratosis lesions, hence the interest in prevention.
